# Distinct ESBL dissemination mechanism associated with the hybrid transposon Tn*1721*/Tn*21* in *bla*_CTX-M-15_-carrying *Salmonella* Enteritidis from poultry in South Korea

**DOI:** 10.1128/spectrum.03755-25

**Published:** 2026-02-12

**Authors:** Junbum Lee, Jin San Moon, Hyokeun Song, Seongbeom Cho

**Affiliations:** 1 College of Veterinary Medicine and Research Institute for Veterinary Science, Seoul National University 26725, Seoul, South Korea; 2 Avian Disease Research Division, Animal and Plant Quarantine Agency 65359, Gimcheon-si, Gyeongsangbuk-do, South Korea; 3 College of Pharmacy, Wonkwang University 201606, Iksan-si, Jeollabuk-do, South Korea; Iowa State University of Science and Technology, Ames, Iowa, USA

**Keywords:** *Salmonella enterica *serovar Enteritidis, antimicrobial resistance, extended-spectrum beta-lactamase, comparative genomics, transposon, poultry

## Abstract

Extended-spectrum beta-lactamase (ESBL)-producing *Salmonella enterica* serovar Enteritidis (*S*. Enteritidis) is emerging as a significant threat to food safety via its limitation of therapeutic options and potential transmission through poultry products. However, the structural and genetic characteristics of mobile genetic elements (MGEs) associated with horizontal transfer of the ESBL gene in *S*. Enteritidis isolates from poultry remain insufficiently characterized. The present study aimed to identify and characterize the ESBL gene and its associated MGEs and to assess their distribution. Whole-genome sequencing was applied to ESBL-producing and non-ESBL-producing isolates in combination with pan-genome analysis, conjugation assays, and comparative genomics using publicly available genomes. Among 17 isolates, 9 were ESBL-producing and all carried *bla*_CTX-M-15_. We observed co-transfer of *bla*_CTX-M-15_, *tetA*, and the IncF plasmid at relatively high frequencies (2.0–5.3 × 10⁻²) in ESBL-producing isolates. Moreover, we identified a hybrid transposon (Tn*1721*/Tn*21*) inserted into IncF plasmids that comprised elements of Tn*21* (*merRTPCADE*, *tniA*, and *urf2*) and Tn*1721* (*tetA*, *tetR*, a DMT-family efflux gene, and a partial *tnpA*), with IS*Ecp1* and *bla_CTX-M-15_* adjacent to the hybrid transposon. Tn*1721*/Tn*21* was prevalent among *bla_CTX-M-15_*-carrying *S*. Enteritidis isolates from South Korea (19/20) but absent in those from other countries (*n* = 9), suggesting geographical variation. This study identified a unique hybrid Tn*1721*/Tn*21* transposon as the dominant MGE in *bla_CTX-M-15_*-carrying *S*. Enteritidis from South Korean poultry, highlighting its potential role in the regional dissemination of antimicrobial resistance. Continued surveillance and targeted intervention in poultry production are warranted to mitigate the spread of ESBL-producing *S*. Enteritidis.

**IMPORTANCE:**

Extended-spectrum beta-lactamase (ESBL)-producing *Salmonella enterica* serovar Enteritidis from poultry represents a growing public health threat due to limited treatment options and the potential for transmission through the food chain. Despite this concern, the mobile genetic elements underlying ESBL gene dissemination remain insufficiently characterized in South Korean poultry-associated *S*. Enteritidis isolates. In this study, we identified a hybrid transposon, Tn*1721*/Tn*21*, embedded within IncF plasmids and linked to *bla*_CTX-M-15_ in *S*. Enteritidis isolates. This association between Tn*1721*/Tn*21* and *bla*_CTX-M-15_ suggests a region-specific mechanism of resistance dissemination that may reflect antimicrobial selective pressure within poultry production systems. These findings highlight the importance of integrated One Health surveillance to mitigate the emergence and spread of antimicrobial resistance across animal and human populations.

## INTRODUCTION

*Salmonella enterica* serovars, particularly non-typhoidal *Salmonella*, are zoonotic foodborne pathogens that pose a significant threat to global food hygiene and are included in the World Health Organization’s list of high-priority bacterial pathogens (1, 2). Among them, *Salmonella enterica* subsp. *enterica* serovar Enteritidis (*S*. Enteritidis) has emerged as a predominant serovar in human infections worldwide (3). Non-typhoidal salmonellosis in humans can cause acute gastroenteritis, bacteremia, and extraintestinal infections affecting multiple organs, and severe cases can lead to hospitalization and even death if appropriate antimicrobial treatment is not applied (4). Of particular concern is the emergence of antimicrobial-resistant *S*. Enteritidis strains, especially those producing extended-spectrum beta-lactamases (ESBLs).

ESBLs are enzymes that can hydrolyze various types of beta-lactam antimicrobials, including the third-generation cephalosporins (5). Owing to their reduced susceptibility to a vast range of antimicrobials, ESBL-producing bacteria pose great challenges to the field of clinical microbiology, restricting treatment options and contributing to therapeutic failures (6). In the case of human salmonellosis caused by *S*. Enteritidis, third-generation cephalosporins are often considered first-line treatment (7). Therefore, the emergence of ESBL-producing *Salmonella* serotypes poses a significant threat to public health. Isolation of ESBL-producing *Salmonella* species was first reported in 1988 (8), and since then, various types (e.g., CTX-M, AmpC, CMY-2) of ESBL-producing *Salmonella* have been reported (9, 10). In addition, there are also reports of ESBL-producing *Salmonella* serotypes being isolated from poultry sources (11). Given that poultry serves as a reservoir for *Salmonella* serotypes and poultry products are one of the main transmission routes to humans (12, 13), the presence of ESBL-producing *S*. Enteritidis in poultry poses a significant threat to food safety.

Dissemination of ESBL genes is primarily mediated by mobile genetic elements (MGEs), such as plasmids, integrons, and transposons (14, 15). Some MGEs carry not only ESBL genes but also other antimicrobial resistance (AMR) determinants, thereby facilitating the co-transfer of multiple resistance genes (14, 16). Therefore, elucidating the structural characteristics of MGEs associated with ESBL genes in *S*. Enteritidis is essential for evaluating and managing the risk of multidrug resistance transmission. However, previous studies on ESBL-producing *S*. Enteritidis have neither characterized the genetic structures of MGEs carrying ESBL genes (17–19) nor analyzed non-ESBL-producing isolates together to determine whether such MGEs represent features exclusive to ESBL-producing isolates (20, 21).

Therefore, this study aimed to (i) identify ESBL genes present in *S*. Enteritidis isolates from poultry in South Korea and determine the MGEs that mediate their horizontal transfer; (ii) characterize the detailed genomic structure and unique genetic contexts of these MGEs; and (iii) assess whether these features are unique to ESBL-producing isolates by comparing them with non-ESBL-producing isolates and previously reported ESBL-producing *S*. Enteritidis. To achieve this aim, whole-genome sequencing (WGS) in combination with pan-genome analysis, conjugation assays, and comparative genomics was applied.

## RESULTS

### AMR phenotypes of *S*. Enteritidis isolates

Among the 17 *S*. Enteritidis isolates tested in this study, 10 were multidrug-resistant (MDR) isolates, showing resistance to more than 3 classes of antimicrobials (Table 1). The remaining seven isolates either showed resistance only against nalidixic acid or nalidixic acid and ceftriaxone and thus were classified as non-MDR isolates. Among the MDR isolates, nine were identified as ESBL-producing *S*. Enteritidis (MDR/ESBL isolates) by the double-disk test. All ESBL isolates were resistant to ampicillin, streptomycin, tetracycline, gentamicin, ceftriaxone, and aztreonam. MDR isolates without ESBL-producing ability and non-MDR isolates were classified as non-ESBL-producing isolates.

**TABLE 1:** AMR profiles and sequence types of *S*. Enteritidis isolates^*e*^^,^^*f*^

AMR patterns	Number of isolates	Source		AMR phenotype	ESBL-producing phenotype
		Chicken	Duck		
NAL	6	2	4	Non-MDR^*a*^	Non-ESBL^*b*^
CRO-NAL	1	0	1	Non-MDR	Non-ESBL
AMP-S-C-NAL	1	1		MDR^*c*^	Non-ESBL
AMP-GEN-S-TE-CRO-CAZ-ATM-CTX-NAL	2	2		MDR	ESBL^*d*^
AMP-GEN-S-TE-CRO-FEP-ATM-CTX-NAL	2	2		MDR	ESBL
AMP-GEN-S-TE-F-CRO-CAZ-ATM-CTX-NAL	2	2		MDR	ESBL
AMP-GEN-S-TE-CRO-CAZ-FEP-ATM-CTX-NAL	3	3		MDR	ESBL
Total	17	12	5		

^*a*^ *S*. Enteritidis isolates showing resistance to less than three antimicrobial classes.

^*b*^ *S*. Enteritidis isolates lacking ESBL-producing ability.

^*c*^ *S*. Enteritidis isolates showing resistance to three or more antimicrobial classes.

^*d*^ ESBL-producing *S*. Enteritidis isolates.

^*e*^ AMR profiles of *S*. Enteritidis isolates. AMR profiles were identified by the disk diffusion method using breakpoints from the CLSI guidelines. ESBL-producing abilities were confirmed by the standard double-disk method.

^*f*^ AMP, ampicillin; ATM, aztreonam; C, chloramphenicol; CAZ, ceftazidime; CLSI, Clinical and Laboratory Standards Institute; CRO, ceftriaxone; CTX, cefotaxime; ESBL, extended-spectrum beta-lactamase; F, nitrofurantoin; FEP, cefepime; GEN, gentamicin; MDR, multidrug-resistant; NAL, nalidixic acid; S, streptomycin; TE, tetracycline.

### Multi-locus sequence types, AMR genes, virulence factors, and plasmids of the *S*. Enteritidis isolates

Multi-locus sequence typing (MLST) results (Table 2) showed that all 17 isolates belonged to sequence type 11 (ST11), regardless of isolated year (2011, 2013, and 2019), source (chicken and duck), sampling site (carcass, feces, and meat), or ESBL-producing and AMR phenotypes. AMR genes were identified by WGS of *S*. Enteritidis isolates (Table 2). All *S*. Enteritidis isolates in this study carried the aminoglycoside resistance gene *aac(6*′*)-Iaa*. Other aminoglycoside resistance genes, including *aph(3*″*)-lb* (*n* = 9), *aph (6)-ld* (*n* = 9), *aac (3)-lld* (*n* = 8), and *aph(3*′*)-la* (*n* = 6), were also detected in the MDR and MDR/ESBL isolates. Mutations in *gyrA* were found in all 17 *S*. Enteritidis isolates, with the substitution being D87N in MDR/ESBL isolates and D87G in MDR and non-MDR isolates. The sulfonamide resistance gene *sul2* was found in eight isolates, seven of which were MDR/ESBL isolates, and one was an MDR isolate. Two isolates carried the beta-lactam gene *bla*_TEM-1B_, while one *bla*_TEM-1B_*-*carrying isolate (SEC-03, an MDR/ESBL isolate) also carried other beta-lactam genes, including *bla*_TEM-104_, *bla*_TEM-198_, *bla*_TEM-217_, and *bla*_TEM-234_. The chloramphenicol resistance gene *catA2* was present in one MDR isolate and one MDR/ESBL isolate. The tetracycline resistance gene *tetA* was found in nine isolates, which were all MDR/ESBL isolates. The ESBL-encoding gene identified was *bla*_CTX-M-15_, which was present in all MDR/ESBL isolates. The AMR genes found in only MDR/ESBL isolates were *bla*_CTX-M-15_ and *tetA*.

**TABLE 2:** Phenotypic and genetic characteristics of *S*. Enteritidis isolates from South Korean poultry^*d*^^,^^*e*^

Isolates	AMR phenotype	MLST	Aminoglycosides	Sulfonamides	Tetracyclines	Beta-lactams	Chloramphenicol	*gyrA* mutation
SED-01	Non-MDR^*a*^	ST11	*aac(6*′*)-laa*					D87G
SED-02	Non-MDR	ST11	*aac(6*′*)-laa*					D87G
SED-03	Non-MDR	ST11	*aac(6*′*)-laa*					D87G
SED-04	Non-MDR	ST11	*aac(6*′*)-laa*					D87G
SED-05	Non-MDR	ST11	*aac(6*′*)-laa*					D87G
SEC-01	MDR^*b*^	ST11	*aac(6*′*)-laa, aph(3*″*)-lb, aph (6)-ld,* *aac (3)-lld*	*sul2*	*tetA*	*bla*_CTX-M-15_		D87N
SEC-02	MDR^*b*^	ST11	*aac(6*′*)-laa, aph(3*″*)-lb, aph (6)-ld, aac (3)-lld, aph(3*′*)-la*	*sul2*	*tetA*	*bla*_CTX-M-15_		D87N
SEC-03	MDR^*b*^	ST11	*aac(6*′*)-laa*, *aph(3*″*)-lb*, *aph (6)-ld*, *aac (3)-lld*, *aph(3*′*)-la*	*sul2*	*tetA*	*bla*_TEM-1B_ *, bla*_TEM-104_ *, bla*_TEM-198_ *, bla*_TEM-217_ *, bla*_TEM-234_ *, bla*_CTX-M-15_	*catA2*	D87N
SEC-04	MDR^*c*^	ST11	*aac(6*′)*-laa*, *aph(3*″*)-lb*, *aph (6)-ld*	*sul2*		*bla*_TEM-1B_	*catA2*	D87G
SEC-05	MDR^*b*^	ST11	*aac(6*′*)-laa*, *aph(3*″*)-lb*, *aph (6)-ld*, *aac (3)-lld*, *aph(3*′*)-la*	*sul2*	*tetA*	*bla*_CTX-M-15_		D87N
SEC-06	MDR^*b*^	ST11	*aac(6*′*)-laa*, *aph(3*″*)-lb*, *aph (6)-ld*, *aac (3)-lld*, *aph(3*′*)-la*	*sul2*	*tetA*	*bla*_CTX-M-15_		D87N
SEC-07	MDR^*b*^	ST11	*aac(6*′*)-laa*, *aph(3*″*)-lb*, *aph (6)-ld*, *aac (3)-lld*, *aph(3*′*)-la*	*sul2*	*tetA*	*bla*_CTX-M-15_		D87N
SEC-08	Non-MDR	ST11	*aac(6*′*)-laa*					D87G
SEC-09	Non-MDR	ST11	*aac(6*′*)-laa*					D87G
SEC-10	MDR^*b*^	ST11	*aac(6*′*)-laa*, *aph(3*″*)-lb*, *aph (6)-ld*, *aac (3)-lld*, *aph(3*′*)-la*	*sul2*	*tetA*	*bla*_CTX-M-15_		D87N
SEC-11	MDR^*b*^	ST11	*aac(6*′*)-laa, aph (6)-ld, aac (3)-lld*		*tetA*	*bla*_CTX-M-15_		D87N
SEC-12	MDR^*b*^	ST11	*aac(6*′*)-laa*, *aac (3)-lld*		*tetA*	*bla*_CTX-M-15_		D87N

^*a*^ *S*. Enteritidis isolates showing resistance to less than three antimicrobial classes.

^*b*^ ESBL-producing isolates. All ESBL-producing isolates were classified as MDR, showing resistance to three or more antimicrobial classes.

^*c*^ *S*. Enteritidis isolates showing resistance to three or more antimicrobial classes but lacking ESBL-producing ability.

^*d*^ AMR phenotypes, sequence types, and detected AMR genes of *S*. Enteritidis isolates. AMR genes were identified using the ResFinder database and are listed according to their antimicrobial drug classes.

^*e*^ AMR, antimicrobial resistance; ESBL, extended-spectrum beta-lactamase; MDR, multidrug-resistant isolate; MLST, Multi-Locus Sequence Type; ST, sequence type.

Plasmid identification results showed that all *S*. Enteritidis isolates carried the replicon sequences of the plasmids IncFIB and IncFII (Table 3). The IncX1 replicon sequence was found in two isolates: one MDR isolate and one MDR/ESBL isolate. The IncQ1 replicon sequence was present in seven MDR/ESBL isolates; among these, five MDR/ESBL isolates also carried the replicon sequences of IncHI2 and IncHI2A. The replicon sequence of Col156 was found in two MDR/ESBL isolates.

**TABLE 3:** Presence of plasmid replicon sequences in the genomes of *S*. Enteritidis isolates^*c*^^,^^*d*^

Isolates	ESBL-producing phenotype	IncFIB	IncFII	IncX1	IncHI2	IncHI2A	IncQ1	Col156
SED-01	Non-ESBL^*a*^	+	+					
SED-02	Non-ESBL	+	+					
SED-03	Non-ESBL	+	+					
SED-04	Non-ESBL	+	+					
SED-05	Non-ESBL	+	+					
SEC-01	ESBL^*b*^	+	+				+	
SEC-02	ESBL	+	+		+	+	+	
SEC-03	ESBL	+	+	+				
SEC-04	Non-ESBL	+	+	+				
SEC-05	ESBL	+	+		+	+	+	
SEC-06	ESBL	+	+		+	+	+	
SEC-07	ESBL	+	+		+	+	+	
SEC-08	Non-ESBL	+	+					
SEC-09	Non-ESBL	+	+					
SEC-10	ESBL	+	+		+	+	+	
SEC-11	ESBL	+	+					+
SEC-12	ESBL	+	+					+

^*a*^ *S*. Enteritidis isolates lacking ESBL-producing ability.

^*b*^ ESBL-producing *S*. Enteritidis isolates.

^*c*^ Presence of plasmid replicon sequences in *S*. Enteritidis isolates identified using PlasmidFinder. The presence of plasmid replicon sequences is indicated as “+,” while absence of replicon sequences is indicated as blank cells.

^*d*^ ESBL, extended-spectrum beta-lactamase.

**Fig 1:**
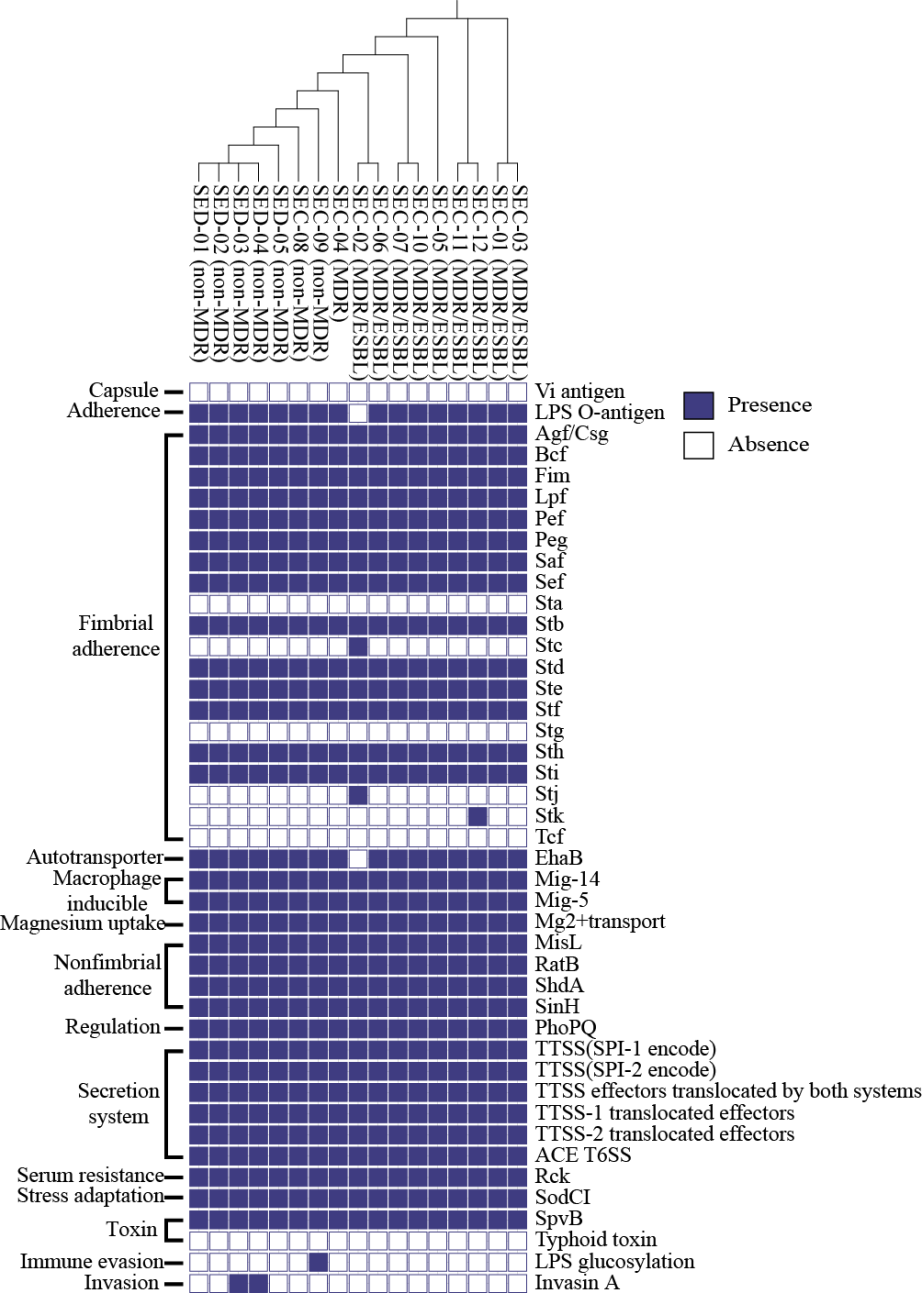
Identification of the presence or absence of virulence genes in 17 *S*. Enteritidis isolates. Heatmap showing the presence or absence of virulence factors found in the genome of *S*. Enteritidis isolates. Virulence factors were identified using The VFanalyzer against the VFDB. The panel on the right indicates the name of virulence factors from the VFDB, and the panel on the left indicates their virulence mechanism. VFDB, virulence factor database.

*Salmonella* pathogenicity islands (SPIs) and virulence genes were identified *in silico* to compare the virulence potential of *S*. Enteritidis isolates. Most isolates shared the same SPIs (SPI-1, SPI-2, SPI-3, SPI-4, SPI-5, SPI-9, SPI-10, SPI-13, SPI-14, CS54_island, and C63PI), regardless of their ESBL-producing abilities (Table S1). A total of 178 virulence genes related to fimbrial and non-fimbrial adherence, macrophage induction, magnesium uptake, two-component system regulation, secretion system, serum resistance, stress adaptation, and toxins were identified among the *S*. Enteritidis isolates (Fig. 1). In particular, all *S*. Enteritidis isolates consistently carried major virulence genes, such as the type three secretion system genes (*invA*, *sipA*, *sipB*, *sipC*, *ssaV*, and *sifA*), toxin gene (*spvB*), and fimbrial adherence determinants (*fimA* and *fimH*).

### Pan-genome analysis and sequence-based comparison between ESBL-producing and non-ESBL-producing isolates

**Fig 2:**
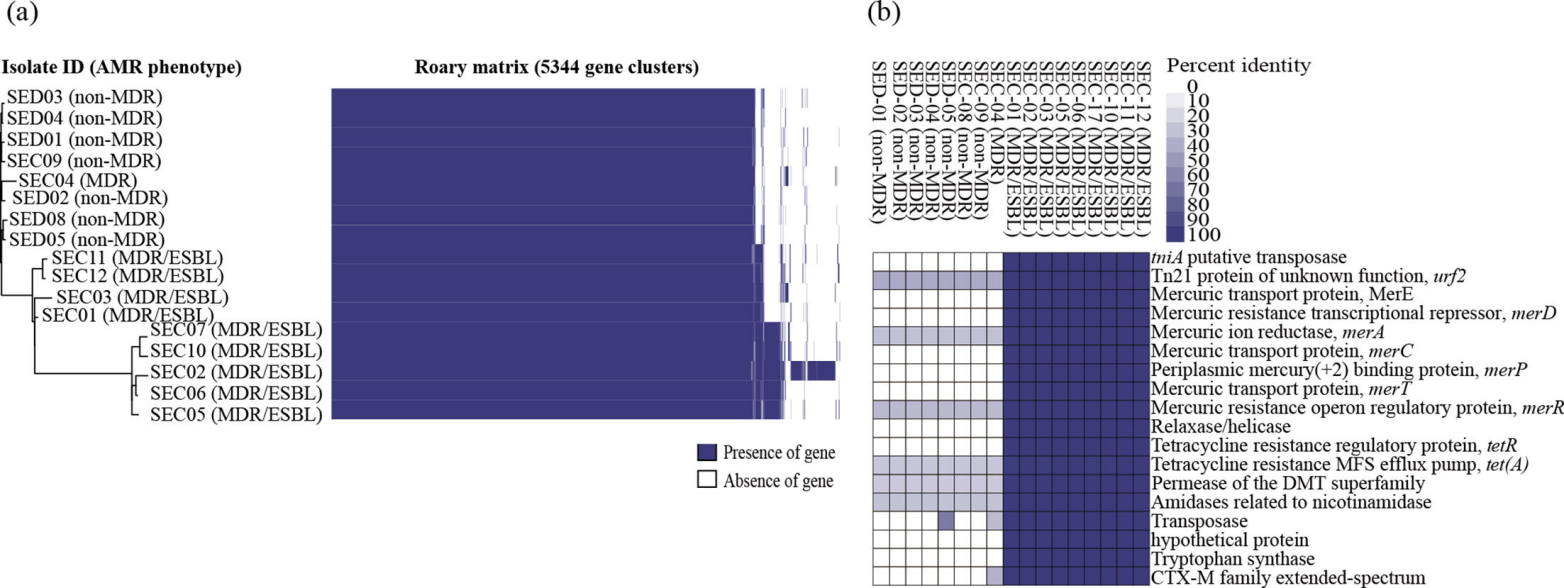
Pan-genome matrix of 17 *S*. Enteritidis isolates and sequence-based comparison of genes unique to MDR/ESBL isolates. (**a**) Heatmap showing the pan-genome of 17 *S*. Enteritidis isolates analyzed using Roary. The phylogenetic tree on the left was constructed based on the presence and absence of genes using Roary. (**b**) Heatmap showing the sequence identity of genes uniquely found in MDR/ESBL isolates compared to non-ESBL-producing isolates. Gene annotation and sequence identity comparison were performed using the Rapid Annotation using Subsystem Technology (RAST) server. MDR, multidrug-resistant isolate; ESBL, extended-spectrum beta-lactamase; MDR/ESBL isolates, ESBL-producing isolates.

The pan-genome of 17 *S*. Enteritidis isolates consisted of 5,344 genes, including 4,408 core genes (identified in 99%–100% of isolates), 342 shell genes (identified in 15%–95% of isolates), and 594 cloud genes (identified in 0%–15% of isolates) (Fig. 2a and Table S2). Although the pan-genome of *S*. Enteritidis comprised a high percentage of core genes, analysis of the presence/absence of genes revealed genes commonly found in all MDR/ESBL *S*. Enteritidis but absent in non-ESBL-producing isolates. Specifically, 64 genes were exclusively present in MDR/ESBL isolates. Among these, 21 were annotated to specific functions, while 43 were not assigned to specific functions and identified as hypothetical proteins.

To investigate the genes unique to MDR/ESBL *S*. Enteritidis isolates (ESBL-unique genes) and to assess their association with MGEs, sequence alignment analysis was performed using the RAST server with isolate SEC07 as the reference. SEC07 was selected as the reference genome because it exhibited the highest assembly quality among MDR/ESBL isolates, as indicated by the highest N50 value and lowest number of contigs (Table S3). Sequence alignment revealed that ESBL-unique genes were present at a high percentage identity in all nine MDR/ESBL isolates but were absent or showed low percentage identity in non-ESBL-producing isolates (Fig. 2b). The ESBL-unique genes identified in the MDR/ESBL isolates included those related to transposons (*tniA*, Tn*21* protein of unknown function *urf2*), mercury resistance (*merRTPCADE*), relaxase/helicase, tetracycline resistance (*tetR* and *tetA*), the drug/metabolite transporter (DMT) superfamily, and tryptophan synthase, along with the ESBL gene from the CTX-M family (*bla*_CTX-M-15_).

### Conjugation assay of the ESBL gene *bla*_CTX-M-15_

A conjugation assay was performed to investigate the transferability of *bla*_CTX-M-15_ from MDR/ESBL isolates. Transmission of the *bla*_CTX-M-15_ gene to the recipient strain was confirmed by the conjugation assay. The conjugation assay further confirmed the co-transfer of the *tetA* gene and the IncF plasmid to the recipient *Escherichia coli* along with the *bla*_CTX-M-15_ gene (Table 4 and Fig. S1). Conjugation frequency ranged from 2 × 10^−2^ to 5.3 × 10^−2^. These results demonstrate that MDR/ESBL isolates can transfer the *bla*_CTX-M-15_ gene to recipient *E. coli* J53 along with the tetracycline resistance gene and IncF plasmid.

**TABLE 4:** Conjugation frequency of *bla*_CTX-M-15_ in MDR/ESBL isolates^*b*^

Isolates	Transconjugants	Recipient	Conjugation frequency^*a*^
SEC-01	4 × 10^5^	1.5 × 10^7^	2.6 × 10^−2^
SEC-02	3 × 10^5^	1.5 × 10^7^	2.0 × 10^−2^
SEC-03	7 × 10^5^	1.5 × 10^7^	4.7 × 10^−2^
SEC-05	6 × 10^5^	1.5 × 10^7^	4.0 × 10^−2^
SEC-06	8 × 10^5^	1.5 × 10^7^	5.3 × 10^−2^
SEC-07	7 × 10^5^	1.5 × 10^7^	4.7 × 10^−2^
SEC-10	4 × 10^5^	1.5 × 10^7^	2.6 × 10^−2^

^*a*^ Conjugation frequency = number of transconjugants (CFU/mL)/number of recipients (CFU/mL).

^*b*^ CFU, colony-forming unit.

### Structural and genetic characteristics of MGEs associated with *bla*_CTX-M-15_

**Fig 3:**
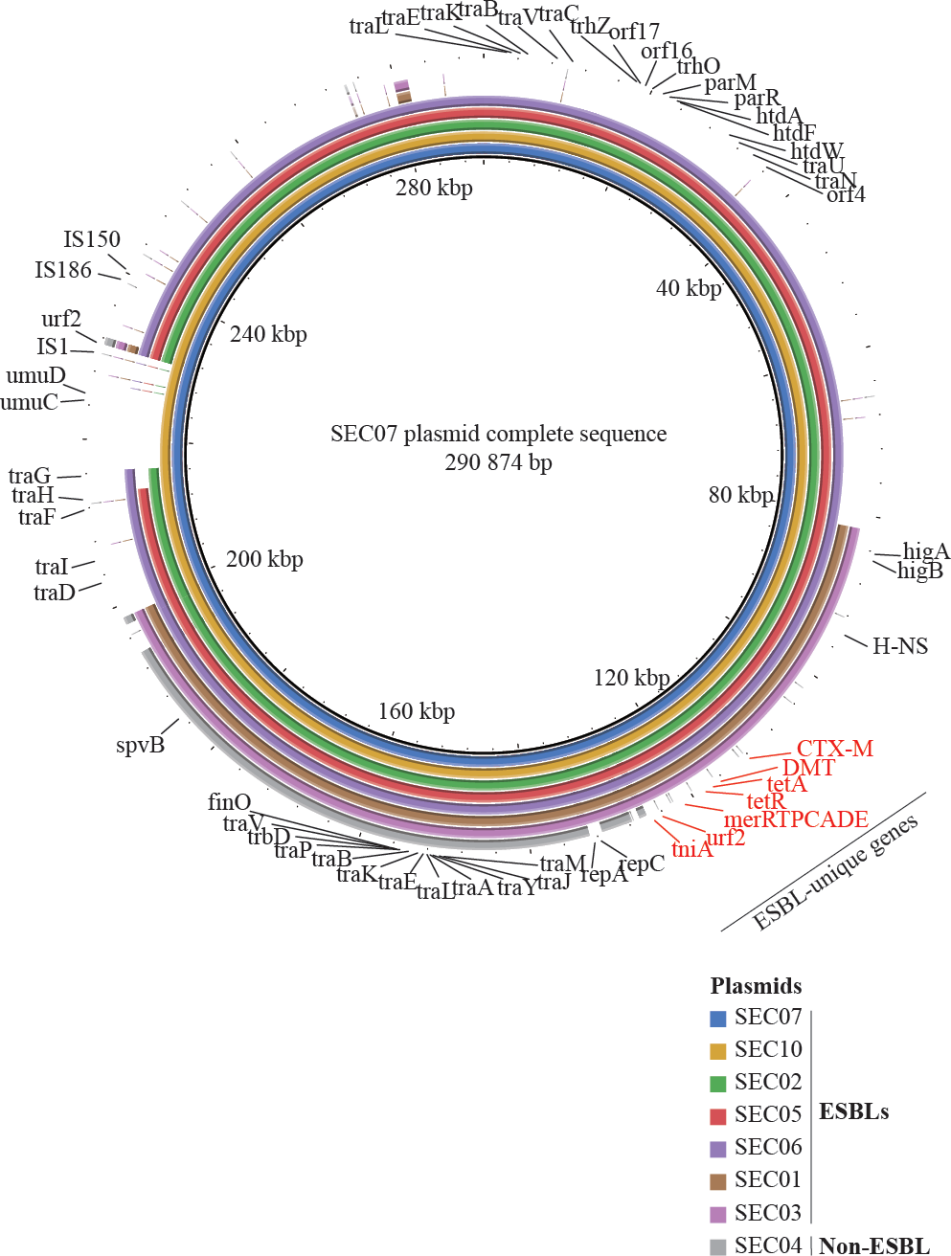
Comparative genomic analysis of complete plasmid sequences among *S*. Enteritidis isolates. The plasmid sequences of six MDR/ESBL and one non-ESBL-producing isolates were aligned and compared against the complete IncF plasmid of SEC07 (290,874 bp) using BRIG (version 0.95). Each circle represents the plasmid from each isolate, with the innermost circle indicating the reference plasmid (SEC07). ESBL, extended-spectrum beta-lactamase; MDR/ESBL, ESBL-producing isolates.

To identify the association between *bla*_CTX-M-15_ and MGEs, hybrid assembly was performed for seven MDR/ESBL isolates and one MDR isolate to obtain complete genome sequences. Plasmid contigs were extracted, and comparative genomic analysis revealed that all *S*. Enteritidis isolates in this study carried the IncF plasmid, which was identified by the presence of genes associated with conjugative transfer of the plasmid (such as *finO*, *traC*, and *traV*) (Fig. 3). The ESBL gene *bla*_CTX-M-15_ was present in all plasmids from the MDR/ESBL isolates, and previously identified ESBL-unique genes were located near *bla*_CTX-M-15_. These plasmids could be grouped into three types. Two MDR/ESBL isolates (SEC01 and SEC03) carried a relatively smaller plasmid of 116,940 bp that contained previously identified ESBL-unique genes (including *bla*_CTX-M-15_, tetracycline resistance genes, and mercury resistance genes). Five MDR/ESBL isolates (SEC02, SEC05, SEC06, SEC07, and SEC10) carried larger IncF plasmids ranging from 277,588 to 293,986 bp in size. The MDR isolate (SEC04), a non-ESBL-producing isolate, also carried an IncF plasmid of 97,007 bp. However, the IncF plasmid from the MDR isolate lacked *bla*_CTX-M-15_ and associated ESBL-unique genes. Virulence factor genes, such as *spvB* or *higB*, were located on the IncF plasmid in both MDR/ESBL and MDR isolates, suggesting the potential for the co-transfer of virulence factors during horizontal gene transfer.

**Fig 4:**
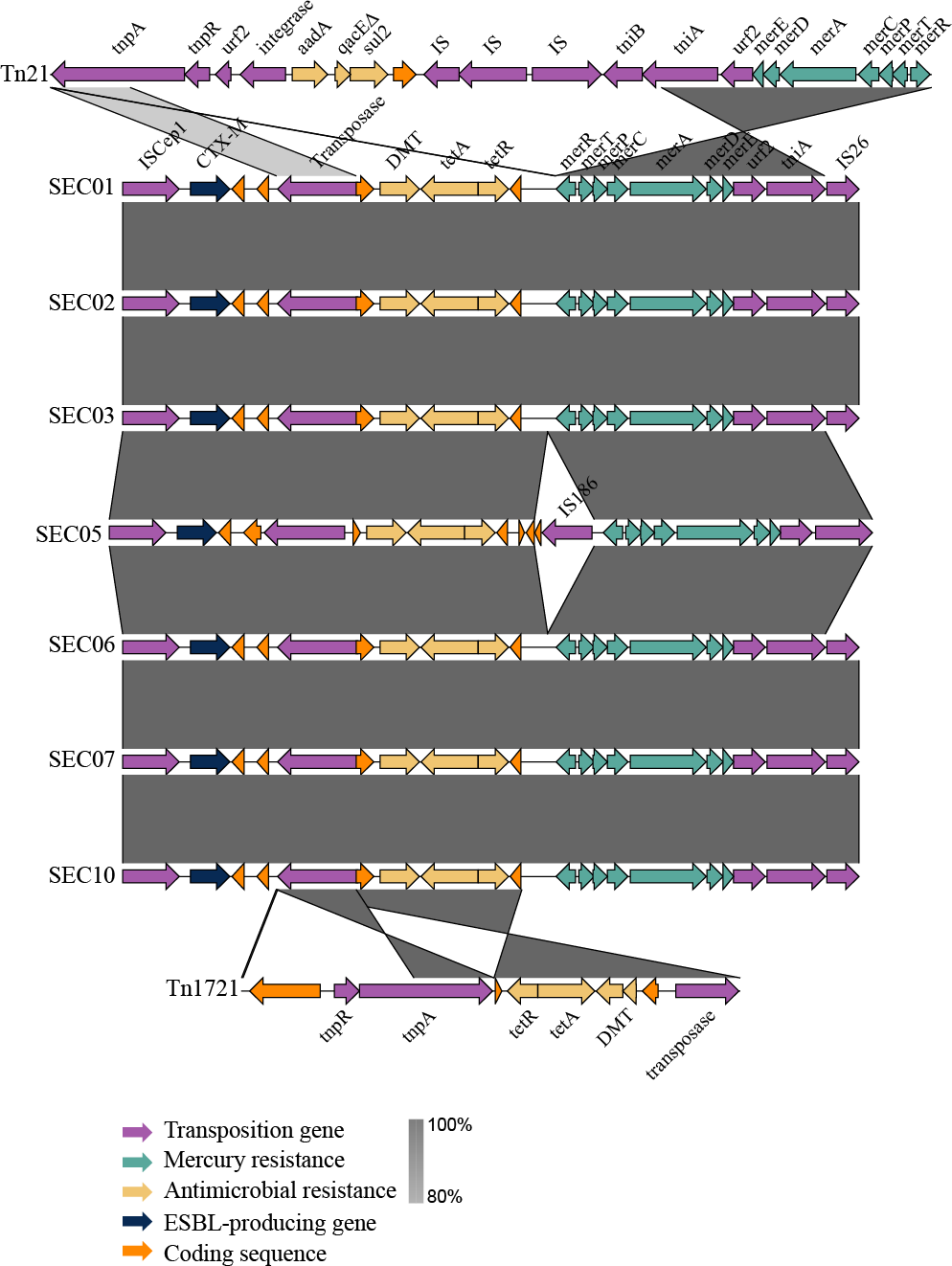
Structural characteristics of the hybrid transposon Tn*1721*/Tn*21* identified in *S*. Enteritidis isolates. Nucleotide sequences of hybrid transposon Tn*1721*/Tn*21* identified in seven MDR/ESBL *S*. Enteritidis isolates were aligned and compared with reference sequences of transposon Tn*21* and Tn*1721* obtained from the NCBI. MDR, multidrug-resistant; ESBL, extended-spectrum beta-lactamase; MDR/ESBL, ESBL-producing isolates; NCBI, National Center for Biotechnology Information.

To investigate the structural characteristics of the MGEs associated with insertion of the ESBL gene into the IncF plasmid, sequences adjacent to *bla*_CTX-M-15_ were analyzed using BLAST to identify transposons or insertion sequences with high sequence similarity (Fig. 4). The BLAST results revealed that the ESBL-unique genes in the MDR/ESBL isolates represented a hybrid transposon composed of elements from both Tn*21* and Tn*1721*. Specifically, the *mer* operon (*merRTPCADE*), *urf2*, and a partial *tniA* gene from Tn*21* showed 100% identity to a partial region of the ESBL-unique genes identified in this study. Meanwhile, genes, such as tetracycline resistance genes (*tetA* and *tetR*) and the DMT superfamily gene showed no homology with Tn*21* but instead exhibited high sequence identity with Tn*1721*. The ESBL gene *bla*_CTX-M-15_ was adjacent to the hybrid transposon Tn*1721*/Tn*21*, along with the insertion sequence IS*Ecp1*.

### Comparative genomic analysis with *bla*_CTX-M-15_*-*carrying *S.* Enteritidis from other studies

**Fig 5:**
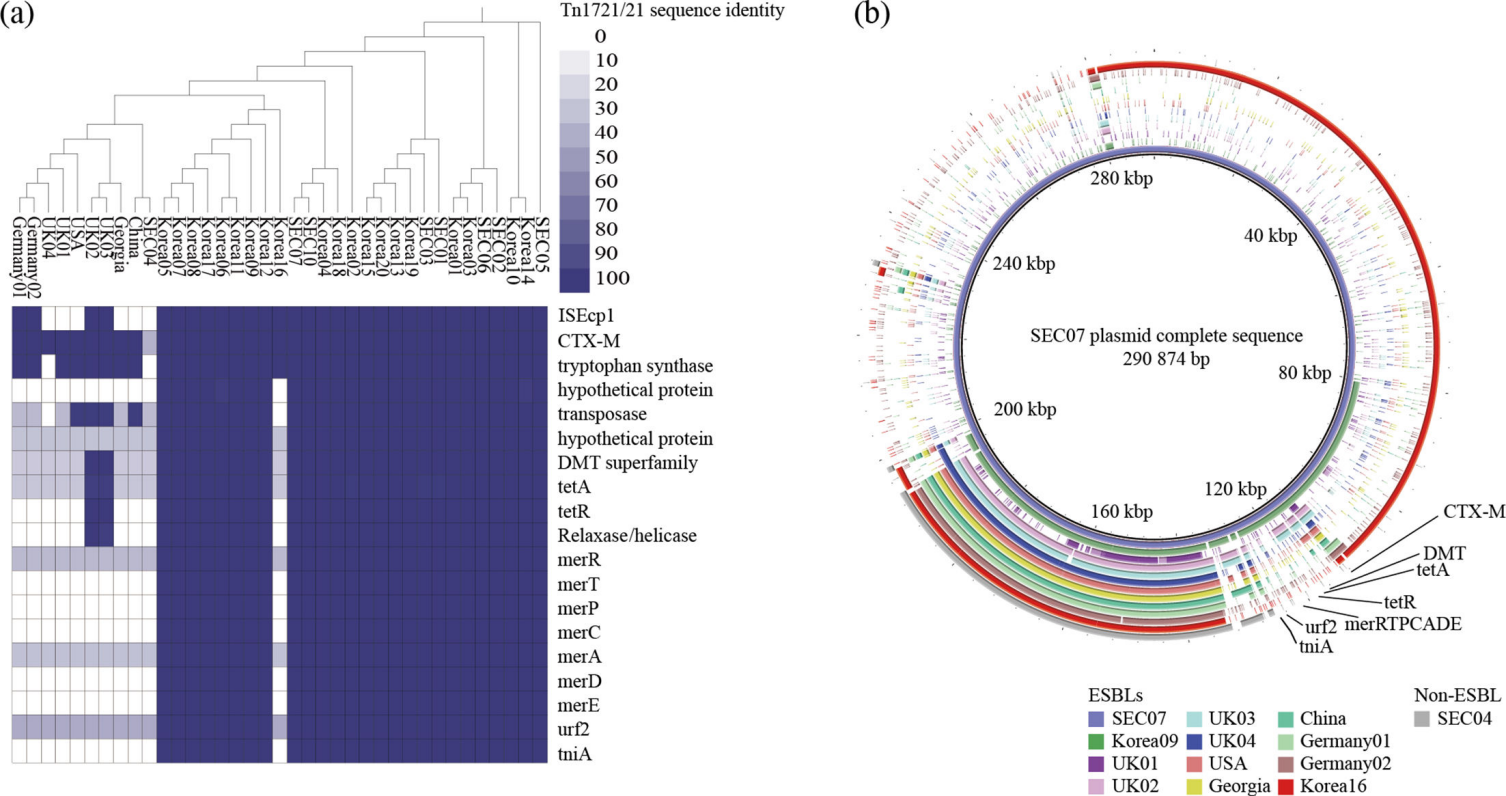
Comparative genomic analysis between *bla*_CTX-M-15_-carrying *S*. Enteritidis isolates. (**a**) Phylogenetic tree based on SNP distances was constructed for the MDR/ESBL and non-ESBL-producing *S*. Enteritidis isolates from this study, along with *bla*_CTX-M-15_-carrying isolates from previous studies. (**b**) Comparative genomics of the IncF plasmid from SEC07 with plasmid sequences from nine *bla*_CTX-M-15_-carrying *S*. Enteritidis isolates from countries outside South Korea, two South Korean isolates (Korea09 and Korea16), and a non-ESBL-producing isolate (SEC04) from this study. Plasmid sequences were aligned and visualized using BRIG. SNP, single-nucleotide polymorphism; MDR, multidrug-resistant; ESBL, extended-spectrum beta-lactamase; MDR/ESBL, ESBL-producing isolates.

Genome assembly data of *S*. Enteritidis isolates carrying the *bla*_CTX-M-15_ gene were retrieved from the National Center for Biotechnology Information (NCBI) database to investigate whether the unique gene sequences of MDR/ESBL isolates from this study were shared with ESBL-producing *S*. Enteritidis isolates from other sources (Fig. 5). Among the 29 isolates from the NCBI database, 20 were from South Korea, and the others were from China (*n* = 1), Germany (*n* = 2), Georgia (*n* = 1), the United Kingdom (*n* = 4), and the United States (*n* = 1). Among the isolates from South Korea, 14 were from chicken meat, and 6 were from chicken-associated environmental sources, including chicken farms and transport trucks. All isolates from countries other than South Korea were of human origin.

Phylogenetic relatedness between these NCBI-obtained isolates and isolates from this study was analyzed based on the single-nucleotide polymorphism distances using SEC07 as the reference isolate (Fig. 5a). SEC07 was used as the reference genome across comparative genomic analyses to maintain consistency. The results revealed that *bla*_CTX-M-15_-carrying *S*. Enteritidis isolates from other countries clustered closer to a non-ESBL-producing isolate from this study (SEC04) than to MDR/ESBL isolates or South Korean isolates. In contrast, *bla*_CTX-M-15_-carrying *S*. Enteritidis isolates from South Korea clustered closer to MDR/ESBL isolates from this study. To investigate the presence of the hybrid transposon Tn*1721*/Tn*21* in previously reported ESBL-producing isolates, genome data obtained from the NCBI database were annotated and aligned against the genome of isolate SEC07 using the RAST server (Fig. 5a). Sequence alignment results revealed that the hybrid transposon Tn*1721*/Tn*21* was also present in *S*. Enteritidis isolates from poultry sources in South Korea (98.82%–100%), excluding one isolate from a chicken transport truck (Korea16). However, in *S*. Enteritidis isolates from other countries, transposon Tn*1721*/Tn*21* genes were either absent or present at low percentage identity compared to those in isolates from poultry sources. Among these isolates, five (UK01, UK04, USA, Georgia, and China) did not carry the insertion sequence IS*Ecp1* adjacent to the *bla*_CTX-M-15_ gene. Two isolates from the UK (UK02 and UK03) carried genes with high sequence identity to the transposase, DMT superfamily gene, and tetracycline resistance genes (*tetA* and *tetR*) from transposon Tn*1721*. To compare the genomic locations of *bla*_CTX-M-15_ and the associated MGEs among *bla*_CTX-M-15_-carrying isolates, plasmid contigs were extracted and aligned against the IncF plasmid of isolate SEC07 (Fig. 5b). In isolates from other countries and one South Korean isolate from a chicken transport truck, the *bla*_CTX-M-15_ gene was located on plasmid contigs; however, we did not detect the hybrid transposon Tn*1721*/Tn*21*.

## DISCUSSION

ESBL-producing *S*. Enteritidis poses a significant threat to global food safety owing to its resistance profile, which limits antimicrobial treatment options and its widespread presence in poultry (22, 23). However, previous efforts to characterize the structural and genetic features of the MGEs responsible for ESBL gene dissemination in *S*. Enteritidis from South Korean poultry are limited, and it remains unclear whether these MGE-related characteristics are also broadly present among ESBL-producing *S*. Enteritidis from other countries. In the present study, WGS and comparative genomics revealed a hybrid transposon uniquely present among ESBL-producing *S*. Enteritidis isolates from South Korean poultry. However, the present study has some limitations, including the relatively small number (*n* = 17) of *S*. Enteritidis isolates, restricting broader result generalization. Future studies involving a larger number of both ESBL-producing and non-ESBL-producing *S*. Enteritidis would help to determine whether these identified features are broadly shared among diverse *S*. Enteritidis isolates.

Our findings revealed that *bla*_CTX-M-15_ is the ESBL gene conferring resistance to cephalosporins in all MDR/ESBL isolates. This gene belongs to the CTX-M beta-lactamase family and has emerged as one of the major ESBL-encoding genes worldwide (24–26). Comparative genomic analysis of AMR genes among 17 *S*. Enteritidis isolates revealed that only MDR/ESBL isolates carried the tetracycline resistance gene *tetA*, whereas *tetA* was absent in all non-ESBL-producing isolates. The co-existence of *bla*_CTX-M-15_ and *tetA* among MDR/ESBL isolates, but their absence in non-ESBL-producing isolates, suggests that a single MGE carrying both resistance genes may have mediated the acquisition of *bla*_CTX-M-15_ in MDR/ESBL isolates. All MDR/ESBL isolates also carried IncF plasmid replicons (IncFII and IncFIB), suggesting the involvement of an IncF-type plasmid in the dissemination of *bla*_CTX-M-15_. Conjugation assays supported this finding by confirming the co-transfer of *bla*_CTX-M-15_ and *tetA* along with the IncF plasmid replicons, indicating that both *bla*_CTX-M-15_ and *tetA* are located on the same IncF plasmid. Herein, the conjugation frequency of MDR/ESBL *S*. Enteritidis isolates was 2.0–5.3 × 10^−2^, consistent with previously reported transfer rates for IncF plasmids in *E. coli* (27). These findings suggest that the co-transfer of *bla*_CTX-M-15_ on IncF plasmids may pose a particularly high risk for the spread of ESBL-producing ability.

Virulence factors, including those encoded on SPIs, are important determinants of pathogenicity in *Salmonella* (28). Here, all *S*. Enteritidis isolates (MDR/ESBLs, MDR, and non-MDR) carried SPI-1 and SPI-2, the two most important SPIs that contribute to the virulence of *Salmonella* species (29). Further, all *S*. Enteritidis isolates in our study consistently carried major virulence genes (such as *invA*, *sipABC*, *ssaV*, *sifA*, *spvB*, *fimA*, and *fimH*) associated with invasion (30), secretion (31), intracellular survival (32), toxin production (33), and adhesion (34). These similar virulence gene profiles observed in ESBL-producing and non-ESBL-producing isolates suggest that the ESBL-producing phenotype in these isolates may be attributable to the acquisition of *bla*_CTX-M-15_ in otherwise virulent strains.

Additionally, our pan-genome analysis revealed that MDR/ESBL isolates possessed a set of genes not found in non-ESBL-producing isolates. These ESBL-unique genes included additional AMR determinants, heavy metal resistance genes, and virulence genes, along with *bla*_CTX-M-15_. Notably, the heavy metal resistance genes identified in the MDR/ESBL isolates were the *mer* operon (*merRTPCADE*), which mediates mercury resistance (35). The *mer* operon is a widespread mode of bacterial protection against mercury (36), which can be toxic to bacteria. Pathogenic bacteria in poultry production environments are frequently exposed to heavy metals and antimicrobial compounds, leading to co-selection for resistance to both, thereby promoting the maintenance and spread of AMR genes (37). In addition to *bla*_CTX-M-15_ and *mer* operon genes, MDR/ESBL isolates uniquely carried tetracycline resistance genes (*tetA* and *tetR*), as well as the DMT superfamily gene, which is a group of efflux pumps that can function as a protection mechanism for bacteria by exporting antimicrobial drugs (38). The MDR/ESBL isolates also carried genes linked to the virulence of *Salmonella* species by mediating survival and proliferation. For instance, the nicotinamidase gene, which plays an important role in the viability and proliferation of some pathogenic bacteria (39), and the gene encoding tryptophan synthase, an enzyme required for biofilm formation in *Salmonella* (40), were detected only in the MDR/ESBL isolates. Furthermore, transposon-associated genes, such as *tniA* and *urf2*, were unique to the MDR/ESBL isolates, suggesting that transposable elements are involved in the acquisition and dissemination of *bla*_CTX-M-15_.

Our comparative genomic analysis of complete plasmid sequences further confirmed that *bla*_CTX-M-15_ was located on the IncF plasmid in MDR/ESBL isolates, consistent with the conjugation assay results. Notably, the non-ESBL-producing MDR isolate also carried the IncF plasmid; however, it lacked the genetic elements of ESBL-associated genes described above (such as *merRTPCADE*, *tetA*, *tniA*, and *urf2*). This finding suggests that the ESBL-associated genetic elements were acquired through insertion into an IncF plasmid already present in *S*. Enteritidis. The ESBL-unique genes identified in this study included those typically found in transposon Tn*21* (41). More specifically, a portion of the ESBL-associated genetic elements in our study corresponded to transposition genes (*tniA* and *urf2*) and the mercury resistance operon (*merRTPCADE*) from transposon Tn*21*. However, other genes within the ESBL-associated genetic elements, such as *tetA*, *tetR*, the DMT efflux pump gene, and a partial *tnpA* transposase, exhibited high sequence identity with transposon Tn*1721*. This suggests that the ESBL-associated genetic elements identified in our study are a hybrid transposon, composed of elements derived from both Tn*21* and Tn*1721*. A similar hybrid Tn*1721*/Tn*21* transposon was reported in an *E. coli* isolate, in which translocation from an IncF plasmid to a cryptic conjugative IncX plasmid was observed, facilitating the formation of a novel MDR plasmid (42). Therefore, the presence of the hybrid transposon Tn*1721*/Tn*21* in *S*. Enteritidis poses a risk for the emergence and dissemination of MDR plasmids carrying *bla*_CTX-M-15_.

Moreover, comparative analysis of publicly available *bla*_CTX-M-15_-carrying *S*. Enteritidis genomes revealed notable geographical differences. The South Korean isolates from the NCBI databases predominantly carried the hybrid Tn*1721*/Tn*21* transposon, identical to that identified in our study. In contrast to the South Korean strains, *bla*_CTX-M-15_-carrying *S*. Enteritidis isolates from other countries (China, Germany, Georgia, UK, and USA) lacked the hybrid Tn*1721*/Tn*21* transposon. Furthermore, five of these foreign isolates did not carry the insertion sequence element IS*Ecp1*, which was consistently found adjacent to the *bla*_CTX-M-15_ gene in our isolates. These findings suggest that different MGEs may be involved in the dissemination of *bla*_CTX-M-15_ in *S*. Enteritidis across different geographical regions and that the Tn*1721*/Tn*21*-inserted IncF plasmid has specifically contributed to the spread of *bla*_CTX-M-15_-carrying *S*. Enteritidis in South Korean poultry.

In South Korea, tetracyclines and penicillins were consistently among the largest-selling antimicrobials for treating food-producing animals between 2008 and 2017 (43, 44). In addition, sales of cephalosporins showed an increasing trend during the same period (43). Sustained high sales of these antimicrobials, which may reflect their intensive use in food-producing animals in South Korea, may have exerted a selective pressure, favoring the persistence and dissemination of Tn*1721*/Tn*21*-inserted IncF plasmid-carrying isolates. Previous reports from South Korea have noted that *bla*_CTX-M-15_ is the dominant ESBL gene in ESBL-producing *S*. Enteritidis and that its horizontal transfer is mediated by IncF plasmids (17–19). However, these studies did not characterize the specific MGEs associated with *bla*_CTX-M-15_ or identify other genes that may be co-transferred with it. Using WGS, we further demonstrated a link between *bla*_CTX-M-15_ and the hybrid Tn*1721*/Tn*21* transposon in *S*. Enteritidis isolates. This suggests that the association between *bla*_CTX-M-15_ and Tn*1721*/Tn*21* transposon, under selective pressure from antimicrobial use, may have contributed to the spread of *bla*_CTX-M-15_-carrying *S*. Enteritidis in South Korean poultry. From a One Health perspective, these findings indicate a potential link between antimicrobial use in poultry and the dissemination of *bla*_CTX-M-15_ in *S*. Enteritidis, raising concern about the potential transmission of AMR *S*. Enteritidis to humans through the food chain. Our findings highlight the importance of a One Health approach to AMR surveillance, notably in the context of foodborne transmission from poultry to humans.

One limitation of the current study is the difference in origin between the *bla*_CTX-M-15_-carrying *S*. Enteritidis isolates. While isolates from countries outside South Korea (lacking the hybrid Tn*1721*/Tn*21* transposon) were derived from human clinical cases, the South Korean isolates originated from poultry. Therefore, it remains unclear whether the observed difference in the presence of the hybrid transposon among *bla*_CTX-M-15_-carrying *S*. Enteritidis strains is attributable to geographical variation or host-specific factors. Further studies examining *bla*_CTX-M-15_-carrying *S*. Enteritidis from a broader range of hosts and geographical areas are required to clarify the role of the Tn*1721*/Tn*21* transposon in various epidemiological contexts.

### Conclusion

The present study elucidated the structural and genetic characteristics of the ESBL gene and its associated MGEs in *S*. Enteritidis isolates from South Korean poultry and evaluated whether these characteristics were also present in publicly available genomes from the NCBI database. All MDR/ESBL isolates in this study carried the *bla*_CTX-M-15_, consistent with the dominance of this ESBL genotype in South Korea. In addition to *bla*_CTX-M-15_, MDR/ESBL isolates also carried a hybrid transposon Tn*1721*/Tn*21* inserted within the IncF plasmid. Conjugation assays confirmed the transferability of the Tn*1721*/Tn*21*-inserted IncF plasmid, highlighting the risk of horizontal spread. Furthermore, comparative genomics indicated that the hybrid Tn*1721*/Tn*21* transposon is a distinctive feature of *bla*_CTX-M-15_-carrying *S*. Enteritidis isolates from South Korea. The presence of Tn*1721*/Tn*21* may have provided a selective advantage to *bla*_CTX-M-15_-carrying *S*. Enteritidis under antimicrobial pressure in South Korean poultry environments. From a One Health perspective, these findings underscore how antimicrobial use in food-producing animals might drive the dissemination of resistance genes, reinforcing the need for integrated surveillance across animal and human sectors.

## MATERIALS AND METHODS

### Bacterial isolation

*S*. Enteritidis isolates from duck were isolated at Seoul National University according to the guidelines from the Korean Food Standards Codex manual (45), whereas isolates from chicken were provided by the Animal and Plant Quarantine Agency of South Korea. For duck isolates, meat samples purchased from a commercial market were rinsed in sterile plastic bags with 45 mL buffered peptone water (BPW) (BD Biosciences, Sparks, MD, USA), then transferred to 50 mL sterile Falcon tubes and incubated overnight at 37°C. Subsequently, 1 mL of the BPW culture was inoculated into 10 mL of tetrathionate (TT) broth (BD Biosciences), and 0.1 mL of the BPW culture was inoculated into 10 mL of Rappaport–Vassiliadis (RV) broth (BD Biosciences). The TT cultures were incubated at 37°C, while the RV cultures were incubated at 42°C for 24 h. Following incubation, the cultures were streaked on xylose lactose tergitol (XLT4) agar (KisanBio, Seoul, Republic of Korea) and incubated for 24 h for colony selection. Colonies suspected of *Salmonella* were transferred onto tryptic soy agar (BD Biosciences) for DNA extraction and PCR analysis. The primer sequences used in the PCR analysis are described in Table S4. *Salmonella* isolates were stocked at −80°C deep freezer in 25% glycerol until antimicrobial susceptibility tests and WGS were conducted.

### Antimicrobial susceptibility testing

*S*. Enteritidis isolate stocks were recovered on Mueller Hinton agar and sub-cultured at least twice prior to antimicrobial susceptibility test. The antimicrobial susceptibility of the *S*. Enteritidis isolates was tested by the disk diffusion method (Kirby-Bauer) and interpreted according to the Clinical and Laboratory Standards Institute (CLSI) guideline M100S 29th edition (2020). *E. coli* ATCC 25922 strain was used as the quality control strain for the disk diffusion assay. A total of 16 antimicrobial disks (BD Bioscience) were tested, including ampicillin (AMP, 10 μg), amoxicillin-clavulanate (AMC, 20/10 μg), gentamicin (GEN, 10 μg), streptomycin (S, 10 μg), azithromycin (AZM, 15 μg), tetracycline (TE, 30 μg), ciprofloxacin (CIP, 5 μg), nalidixic acid (NAL, 30 μg), trimethoprim-sulfamethoxazole (SXT, 23.75/1.25 μg), chloramphenicol (C, 30 μg), nitrofurantoin (F, 300 μg), ceftriaxone (CRO, 30 μg), ceftazidime (CAZ, 30 μg), cefepime (FEP, 30 μg), aztreonam (ATM, 30 μg), and cefoxitin (FOX, 30 μg).

*S*. Enteritidis isolates that showed a zone diameter of ≤22 mm for ceftazidime, or ≤27 mm for aztreonam, or ≤25 mm for ceftriaxone were further subjected to a standard ESBL/AmpC double-disk test following CLSI guidelines. Disks of cefotaxime (CTX, 30 μg), ceftazidime, cefotaxime-clavulanate (CTX-CLA, 30/10 μg), and ceftazidime-clavulanate (CAZ-CLA, 30/10 μg) (BD Bioscience) were used to confirm ESBL-producing abilities. MDR *S*. Enteritidis was defined as isolates resistant to three or more antimicrobial classes tested (46). Isolates that did not meet the criteria for either ESBL production or MDR were classified as non-MDR isolates.

### WGS

Genomic DNA was extracted using the NucleoSpin Microbial DNA Kit (Macherey-Nagel, Düren, Germany) according to the manufacturer’s instructions. The DNA library was prepared using TruSeq Nano DNA LT Library Prep Kit (Illumina, Inc., San Diego, CA, USA). The quality of constructed libraries was assessed using 2100 Bioanalyzer System (Agilent Technologies, Santa Clara, CA, USA). Then the DNA libraries from *Salmonella* isolates were sequenced with a 2 × 150 bp read length using NextSeq 500 Sequencing System (Illumina, Inc.). Raw short-read sequences were assembled using SPAdes version 3.14.0 (with default settings) (47).

For additional long-read sequencing, genomic DNA was extracted using either the MagAttract HMW DNA Kit (Qiagen, Venlo, Netherlands) or Quick-DNA HMW MagBead Kit (Zymo Research, Irvine, CA, USA), following the manufacturer’s instructions. To generate libraries, 3 µg of genomic DNA was sheared using the g-tube (Covaris, Woburn, MA, USA), and then small fragments were removed using AMPure PB beads (Pacific Biosciences, Menlo Park, CA, USA). The SMRTbell library was constructed using SMRTbell Prep Kit 3.0 (Pacific Biosciences), and libraries below 5 kb were removed using AMPure PB beads. The sequencing primer was annealed to the SMRTbell template, and DNA polymerase was bound to the complex using the Revio Polymerase Kit (Pacific Biosciences). The long-read sequencing from the SMRTbell library was sequenced using the Revio Sequencing platform (Pacific Biosciences). Raw long reads were assembled together with short reads using Unicycler version 0.5.1 (with default settings) (48).

### Analysis of *S*. Enteritidis WGS data

WGS data were analyzed using the Center for Genomic Epidemiology (CGE) website (https://www.genomicepidemiology.org/). Serotypes of *S*. Enteritidis were confirmed using the SeqSero software version 1.2 (with default settings). MLST was determined from the assembled genome using MLST version 2.0.9 (with minimum depth for an allele of 5×) (49–55). AMR genes within the assembled genomes were detected using ResFinder 4.1 version (with thresholds for percent identity of 90% and a minimum length coverage of 60%) (55, 56). SPIs were identified using SPIfinder 2.0 version (with a percent identity threshold of 95% and a minimum gene length threshold of 60%) (57), and virulence genes were detected using VFanalyzer from the virulence factor database web (58) (https://www.mgc.ac.cn/VFs/). For gene prediction and pan-genome analysis, Prokka version 1.12 (with e-value cut-off of 1e−06) (59) and Roary version 3.12.0 (with minimum blast percentage of 95%) (60) were used. Genes present in 99% of isolates were defined as core genes. Sequence-based alignment and comparative analysis of *S*. Enteritidis genomes were performed using the RAST server and SeedViewer with the RASKtk pipeline and default settings (61–63). Assembly data of 29 *S*. Enteritidis strains were retrieved from the NCBI pathogen isolate browser (accessed on 25 September 2024), using the following criteria: Serovar; Enteritidis, *S*. Enteritidis and AMR genotypes; *bla*_CTX-M-15_. Additional information on these isolates is provided in Table S5 (64–67). Phylogenetic relatedness was analyzed using the CSIPhylogeny version 1.4 from CGE (with default settings) (68). Plasmid contigs were identified from assembled contigs using PLASMe version 1.1 (with minimum coverage of 90%, minimum identity of 90%, and minimum probability of transformer 50%) (69). Figures were generated using Interactive Tree Of Life version 6 (70), Easyfig version 2.2.25 (71), and BRIG (72).

### Conjugation assay

Conjugation assays were conducted using a previously described method with slight modifications (73). Briefly, *E. coli* J53-AziR was used as the recipient strain, and MDR/ESBL *S*. Enteritidis isolates were used as donors. Luria–Bertani agar (BD Biosciences) plates containing 4 mg/L cefotaxime (Sigma-Aldrich, St. Louis, MO, USA) and 100 mg/L sodium azide (Sigma-Aldrich) were used to select transconjugant colonies. The presence of ESBL genes, tetracycline resistance genes, and plasmid replicon types in the transconjugant strains was confirmed using PCR. Primer sequences and reaction conditions used for the conjugation assay are provided in Table S4.

## Data Availability

The sequence data generated in this study have been deposited in the National Center for Biotechnology Information Sequence Read Archive (accession number PRJNA1225312).

## SUPPLEMENTAL MATERIAL

Fig. S1 spectrum.03755-25-s0001.pdf Agarose gel electrophoresis results of PCR products confirming the horizontal transfer of antimicrobial resistance genes and plasmid replicon sequences in transconjugants following the conjugation assay.

Table S1 spectrum.03755-25-s0002.xlsx Salmonella pathogenicity islands of the *S. Enteritidis* isolates.

Table S2 spectrum.03755-25-s0003.xlsx Gene presence and absence table in the pan-genome of *S. Enteritidis* isolates.

Table S3 spectrum.03755-25-s0004.docx Genome assembly quality metrics of MDR/ESBL isolates.

Table S4 spectrum.03755-25-s0005.docx Oligonucleotide sequence of primers used to detect *S. Enteritidis* and antimicrobial resistance genes.

Table S5 spectrum.03755-25-s0006.xlsx Assembly accession numbers of whole genome sequence (WGS) data of *bla*_CTX-M-15_-carrying isolates registered that were used in this study.

ASM does not own the copyrights to Supplemental Material that may be linked to, or accessed through, an article. The authors have granted ASM a non-exclusive, world-wide license to publish the Supplemental Material files. Please contact the corresponding author directly for reuse.
