# Supplementary material for: Distinct ESBL dissemination mechanism associated with the hybrid transposon Tn1721/Tn21 in blaCTX-M-15-carrying Salmonella Enteritidis from poultry in South Korea
Source: Microbiol Spectr. 2026 Feb 12;14(3):e03755-25. doi: 10.1128/spectrum.03755-25 (PMC12955435; doi:10.1128/spectrum.03755-25)
Supplement: Fig. S1 — Agarose gel electrophoresis results of PCR products confirming the horizontal transfer of antimicrobial resistance genes and plasmid replicon sequences in transconjugants following the conjugation assay. [file spectrum.03755-25-s0001.pdf]

(a)

2025-03-07 *blaCTX-M-15* (Raw 1-D Image)

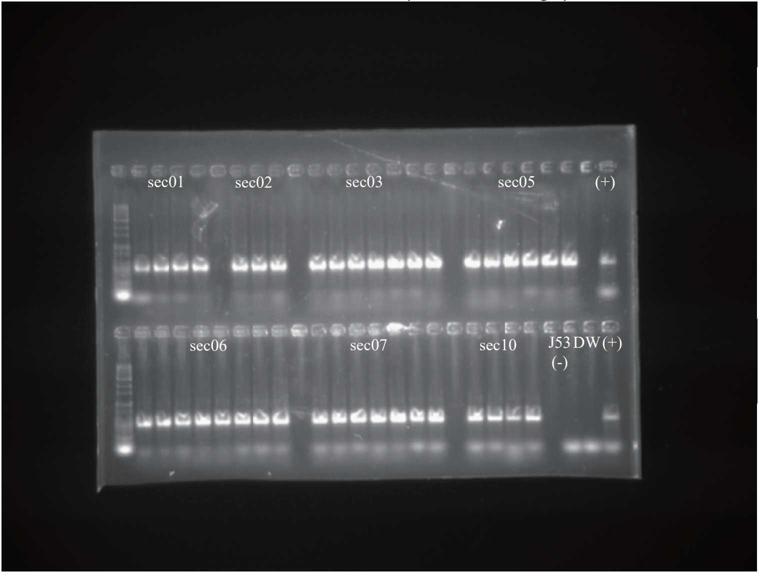

(b)

2025-03-07 tet(A) (Raw 1-D Image)

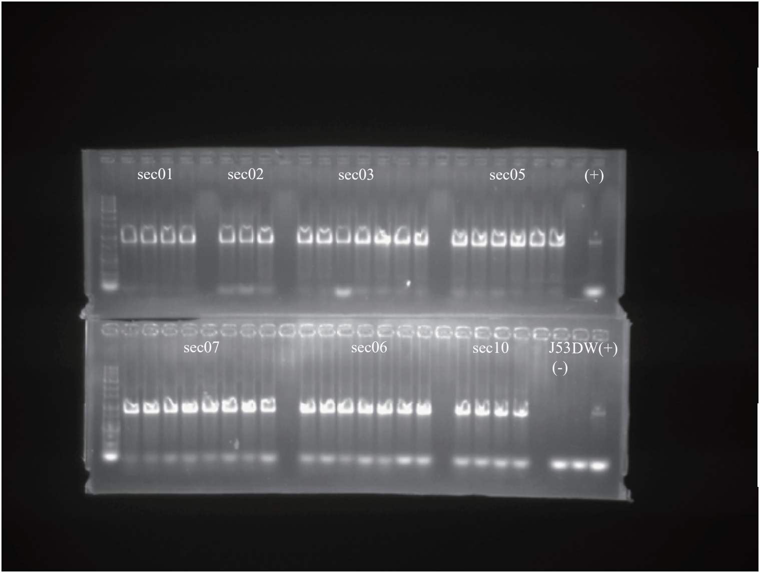

(c)

2025-03-07 IncF plasmid (Raw 1-D Image)

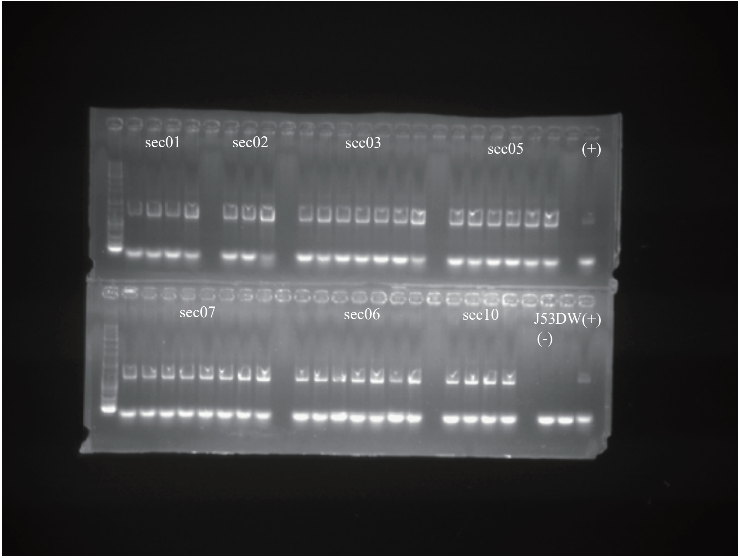

Figure S1. Agarose gel electrophoresis results of PCR products confirming the horizontal transfer of antimicrobial resistance (AMR) genes and plasmid replicon sequences in transconjugants following the conjugation assay. PCR was performed to detect (a) *bla*$_{CTX\text{-}M\text{-}15}$, (b) *tetA*, and (c) the IncF plasmid replicon sequence. The left panel indicates the DNA ladder, and the right panel represents the positive control for each gene. *Escherichia coli* J53 without conjugation and distilled water were used as the negative controls. Donor *S.* Enteritidis isolates corresponding to each transconjugant are labeled.
