# Supplementary material for: Distinct ESBL dissemination mechanism associated with the hybrid transposon Tn1721/Tn21 in blaCTX-M-15-carrying Salmonella Enteritidis from poultry in South Korea
Source: Microbiol Spectr. 2026 Feb 12;14(3):e03755-25. doi: 10.1128/spectrum.03755-25 (PMC12955435; doi:10.1128/spectrum.03755-25)
Supplement: Table S3 — Genome assembly quality metrics of MDR/ESBL isolates. [file spectrum.03755-25-s0004.docx]

Table S3. Genome assembly quality metrics of MDR/ESBL isolates

| Isolates | Number of Contigs | Total length (bp) | GC (%) | N50 (bp) | L50 |
| --- | --- | --- | --- | --- | --- |
| SEC-01 | 118 | 4,572,166 | 52.13 | 433,261 | 4 |
| SEC-02 | 450 | 4,327,353 | 51.65 | 137,432 | 11 |
| SEC-03 | 101 | 4,501,260 | 52.07 | 406,064 | 4 |
| SEC-05 | 93 | 4,728,721 | 51.89 | 478,531 | 3 |
| SEC-06 | 100 | 4,608,206 | 51.87 | 291,722 | 7 |
| SEC-07 | 89 | 4,608,356 | 51.86 | 478,822 | 3 |
| SEC-10 | 261 | 4,578,504 | 51.86 | 305,478 | 5 |
| SEC-11 | 166 | 4,412,142 | 52.13 | 220,652 | 8 |
| SEC-12 | 263 | 4,545,833 | 52.12 | 275,197 | 4 |

Genome assembly quality metrics of ESBL-producing *S*. Enteritidis isolates (MDR/ESBL isolates) from this study were assessed using the Quality Assessment Tool (QUAST)(1).

1. Gurevich A, Saveliev V, Vyahhi N, Tesler G. 2013. QUAST: quality assessment tool for genome assemblies. Bioinformatics 29:1072-1075.
