# Supplementary material for: Distinct ESBL dissemination mechanism associated with the hybrid transposon Tn1721/Tn21 in blaCTX-M-15-carrying Salmonella Enteritidis from poultry in South Korea
Source: Microbiol Spectr. 2026 Feb 12;14(3):e03755-25. doi: 10.1128/spectrum.03755-25 (PMC12955435; doi:10.1128/spectrum.03755-25)
Supplement: Table S4 — Oligonucleotide sequence of primers used to detect S. Enteritidis and antimicrobial resistance genes. [file spectrum.03755-25-s0005.docx]

Table S4. Oligonucleotide sequence of primers used to detect S. Enteritidis and antimicrobial resistance genes

| Target | Nucleotide sequence | Annealing temp. (℃) | Reference |
| --- | --- | --- | --- |
| *S*. Enteritidis  identification | F AGATTGGGCACTACACGTGT | 60 | (1) |
|  | R TGTACTCCACCAGGTAATTG |  |  |
| *blaCTX-M-1* | F GACGATGTCACTGGCTGAGC | 55 | (2) |
|  | R AGCCGCCGACGCTAATACA |  |  |
| *tet(A)* | F GTAATTCTGAGCACTGTCGC | 60 | (3) |
|  | R CTGCCTGGACAACATTGCTT |  |  |
| *IncF* | F TGCTTTTATTCTTAAACTATCCAC | 60 | (4) |
|  | R CTCCCGTCGCTTCAGGGCATT |  |  |

Reference

1. Wang S-J, Yeh D-B, Wei C-I. 2009. Specific PCR primers for the identification of Salmonella enterica serovar Enteritidis in chicken-related samples. Journal of Food and Drug Analysis 17:9.

2. Denagamage TN, Wallner-Pendleton E, Jayarao BM, Xiaoli L, Dudley EG, Wolfgang D, Kariyawasam S. 2019. Detection of CTX-M-1 extended-spectrum beta-lactamase among ceftiofur-resistant Salmonella enterica clinical isolates of poultry. Journal of Veterinary Diagnostic Investigation 31:681-687.

3. Lucarelli C, Dionisi AM, Torpdahl M, Villa L, Graziani C, Hopkins K, Threlfall J, Caprioli A, Luzzi I. 2010. Evidence for a second genomic island conferring multidrug resistance in a clonal group of strains of Salmonella enterica serovar Typhimurium and its monophasic variant circulating in Italy, Denmark, and the United Kingdom. Journal of Clinical Microbiology 48:2103-2109.

4. Villa L, García-Fernández A, Fortini D, Carattoli A. 2010. Replicon sequence typing of IncF plasmids carrying virulence and resistance determinants. Journal of Antimicrobial Chemotherapy 65:2518-2529.
